# 1101. Antiviral Prescription in Children with Influenza in Emergency Departments (ED): New Vaccine Surveillance Network (NVSN), 2016–2020

**DOI:** 10.1093/ofid/ofad500.074

**Published:** 2023-11-27

**Authors:** Tess Stopczynski, Justin Z Amarin, James W Antoon, Olla Hamdan, Laura S Stewart, James Chappell, Andrew J Spieker, Eileen J Klein, Janet A Englund, Geoffrey A Weinberg, Peter G Szilagyi, Robert Hickey, Marian G Michaels, Flor M Munoz, Julie A Boom, Mary A Staat, Elizabeth P Schlaudecker, Jennifer E Schuster, Rangaraj Selvarangan, Angela P Campbell, Heidi L Moline, Veronica Burkel, Samantha M Olson, Natasha B Halasa

**Affiliations:** Vanderbilt University Medical Center, Nashville, TN; Vanderbilt University Medical Center, Nashville, TN; Vanderbilt University Medical Center, Nashville, TN; Vanderbilt University Medical Center, Nashville, TN; Vanderbilt University Medical Center, Nashville, TN; Vanderbilt University Medical Center, Nashville, TN; Vanderbilt University Medical Center, Nashville, TN; University of Washington School of Medicine, Seattle, Washington; Seattle Children’s Hospital, Seattle, Washington; University of Rochester School of Medicine & Dentistry, Rochester, NY; UCLA School of Medicine, Agoura Hills, California; Childrens Hospital of Pittsburgh, Pittsburgh, Pennsylvania; UPMC Children's Hospital of Pittsburgh, Pittsburgh, Pennsylvania; Baylor College of Medicine, Houston, TX; Texas Children’s Hospital, Houston, Texas; Cincinnati Children’s Hospital Medical Center, Cincinnati, Ohio; Cincinnati Children's Hospital Medical Center, Cincinnati, Ohio; Children’s Mercy Kansas City, Kansas City, Missouri; Children’s Mercy Kansas City, Kansas City, Missouri; CDC, Atlanta, GA; Centers for Disease Control and Prevention, Atlanta, Georgia; Eagle Health Analytics / CDC, Atlanta, Georgia; Centers for Disease Control and Prevention, Atlanta, Georgia; Vanderbilt University Medical Center, Nashville, TN

## Abstract

**Background:**

Influenza virus infections are common in children and lead to numerous ED visits each year. The Infectious Diseases Society of America, American Academy of Pediatrics, and the Centers for Disease Control and Prevention recommend outpatient antiviral treatment for children at increased risk of severe influenza illness (e.g., those < 5 years old, but especially < 2 years, and those with certain underlying medical conditions) and recommend treatment within 48-hours of symptom onset. We describe antiviral prescription in children presenting to the ED with influenza from 2016-2020.

**Methods:**

We analyzed data from NVSN, a prospective seven-site acute respiratory illness surveillance study. We enrolled children presenting to the ED with confirmed influenza by research molecular testing, stratified by children at increased risk of severe influenza and with symptom onset ≤ 48-hours. We compared characteristics of children who were prescribed antivirals to those who were not. We used logistic regression to compare the odds of prescription, adjusting for age, gastrointestinal symptoms, symptom duration, underlying conditions, clinical testing before antiviral prescription, and site.

**Results:**

Of the 16,822 children presenting in the ED and tested for influenza, 2,387 (14%) tested positive. Among influenza positive cases, 1,873 (79%) were defined as children at increased risk of severe influenza, and 571 (30%) were prescribed an antiviral. Additionally, there were 281 (12%) children with symptom onset ≤ 48-hours, and only 70 (25%) were prescribed an antiviral (**Table**). Odds of prescription were higher for those clinically tested for influenza and for those with underlying respiratory and cardiovascular conditions, and lower for those who were younger, presented with diarrhea, and had longer duration since symptom onset (**Figure**).

Table 1

**Figure:**
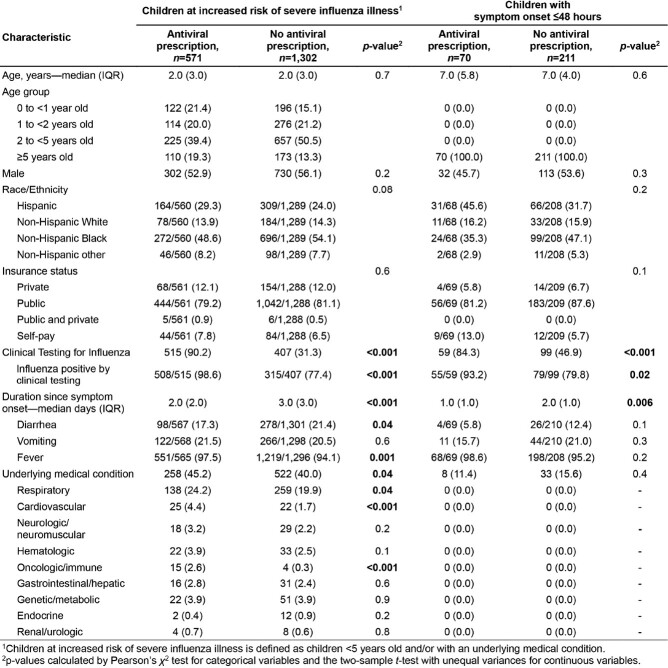

Demographic and clinical characteristics of children with influenza, stratified by risk of severe influenza illness and antiviral prescription.

Figure

**Figure:**
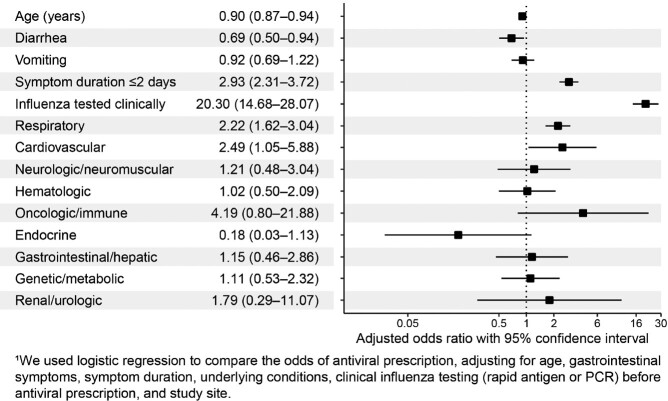

Adjusted odds ratios of antiviral prescription among children with influenza.

**Conclusion:**

While clinical testing and duration since symptom onset predicted antiviral prescription in both children at increased risk of severe influenza and with symptom onset ≤ 48-hours, 70% of children at increased risk were not prescribed an antiviral. Further investigation into clinician perception of antiviral use in children could help identify barriers to antiviral prescription and time-to-treatment.

**Disclosures:**

**Janet A. Englund, MD**, Ark Biopharma: Advisor/Consultant|AstraZeneca: Advisor/Consultant|AstraZeneca: Grant/Research Support|GlaxoSmithKline: Grant/Research Support|Meissa Vaccines: Advisor/Consultant|Merck: Grant/Research Support|Moderna: Advisor/Consultant|Moderna: Grant/Research Support|Pfizer: Advisor/Consultant|Pfizer: Grant/Research Support|Sanofi Pasteur: Advisor/Consultant **Geoffrey A. Weinberg, MD**, Merck & Co: Honoraria **Marian G. Michaels, MD, MPH**, Merck: Grant/Research Support|Viracor: Grant/Research Support **Flor M. Munoz, MD, MSc**, CDC respiratory virus surveillance: Grant/Research Support|Gilead: Grant/Research Support|Moderna, sanofi, aztra zeneca, Merck, GSK: Advisor/Consultant|NIH: DSMB|NIH COVID-19 vaccines in pregnancy: Grant/Research Support|Pfizer Pediatric COVID-19 vaccines: Grant/Research Support|Pfizer, Dynavax, Monderna, Meissa, NIH: DSMB **Mary A. Staat, MD, MPH**, CDC: Grant/Research Support|Cepheid: Grant/Research Support|Merck: Grant/Research Support|NIH: Grant/Research Support|Pfizer: Grant/Research Support|Up-To-Date: Honoraria **Elizabeth P. Schlaudecker, MD, MPH**, Pfizer: Grant/Research Support|Sanofi Pasteur: Advisor/Consultant **Rangaraj Selvarangan, BVSc, PhD, D(ABMM), FIDSA, FAAM**, Abbott: Honoraria|Altona Diagnostics: Grant/Research Support|Baebies Inc: Advisor/Consultant|BioMerieux: Advisor/Consultant|BioMerieux: Grant/Research Support|Bio-Rad: Grant/Research Support|Cepheid: Grant/Research Support|GSK: Advisor/Consultant|Hologic: Grant/Research Support|Lab Simply: Advisor/Consultant|Luminex: Grant/Research Support **Natasha B. Halasa, MD, MPH**, Merck: Grant/Research Support|Quidell: Grant/Research Support|Quidell: donation of kits|Sanofi: Grant/Research Support|Sanofi: vaccine support

